# Advances in the Study of Protein Deamidation: Unveiling Its Influence on Aging, Disease Progression, Forensics and Therapeutic Efficacy

**DOI:** 10.3390/proteomes13020024

**Published:** 2025-06-05

**Authors:** Sunil S. Adav

**Affiliations:** School of Materials Science and Engineering, Nanyang Technological University, 50 Nanyang Avenue, Singapore 639798, Singapore; ssadav@ntu.edu.sg

**Keywords:** deamidation, aging, cancer, dementia, forensics, biopharmaceuticals

## Abstract

Protein deamidation, a nonenzymatic post-translational modification that converts asparagine and glutamine residues into their acidic forms, such as aspartic acid, iso-aspartic acid, or glutamic acid, has emerged as a pivotal process affecting protein stability and function. Once considered a minor biochemical occurrence, deamidation is now recognized for its significant role in aging, age-associated diseases, disease progression, cancer, and therapeutic efficacy. This review explores the recent advances in understanding protein deamidation, its impact on cellular homeostasis, protein misfolding, and age-related and chronic diseases including neurodegeneration and cancer. The study also highlights the challenges posed by deamidation in biopharmaceuticals, where it compromises therapeutic stability and efficacy. Advancements in state-of-the-art analytical techniques and computational approaches for identifying deamidation sites and predicting deamidation-prone regions are discussed, along with deeper insights into how deamidation affects protein structure and function. Based on the current insights, this review underscores the dual role of deamidation as both a natural regulatory process and a contributor to pathological states, providing a roadmap for future research in aging biology, disease mechanisms, and therapeutics.

## 1. Introduction

Protein deamidation is a spontaneous, irreversible post-translational modification (PTM) that has gained considerable attention for its role in cellular aging, disease progression, and the efficacy of therapeutic proteins. It involves the nonenzymatic conversion of amide groups in the side chains of asparagine (Asn) and glutamine (Gln) residues into their corresponding acids like aspartic acid or isoaspartic acid and glutamic acid. Deamidation is also commonly categorized as a degenerative protein modification (DPM) [1]. It was reported over half a century ago [2] and was initially regarded as a relatively harmless and inevitable aspect of protein turnover. Between 1970 and 2000, Robinson’s lab made significant contributions to understanding the Asn and Gln deamidation rates, primarily in peptides. Their comprehensive studies [3,4,5,6,7,8] laid the foundation for the field, alongside other key contributions. The pioneering work, particularly by Arthur Robinson and Noah Robinson, revealed its broader biological significance.

**Figure 1:**
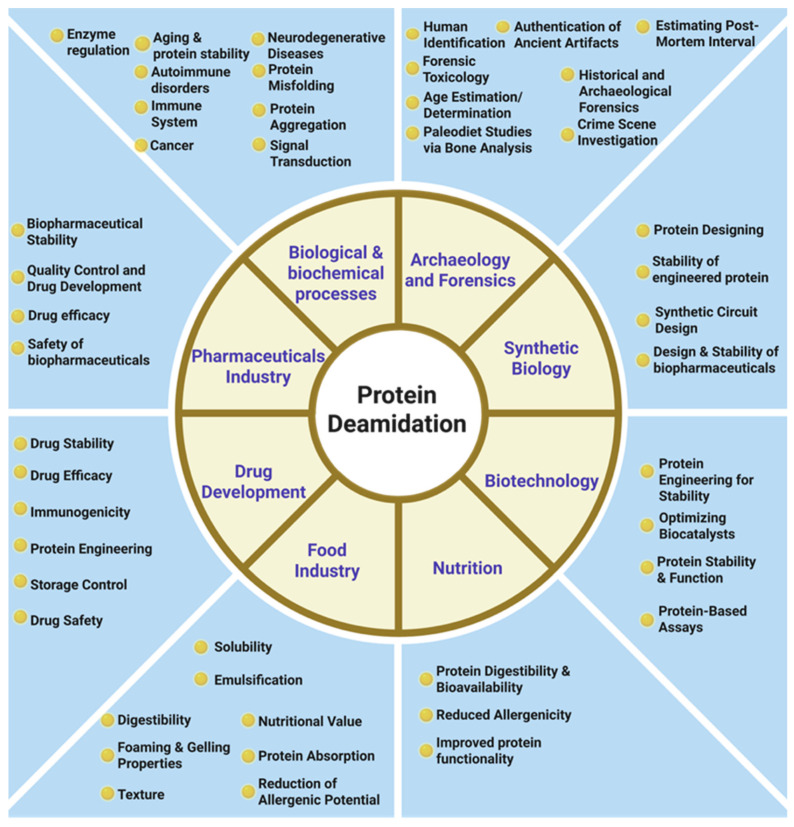
Biological and societal outreach of protein deamidation. Applications of protein deamidation across various disciplines.

Recent advancements in molecular biology and analytical techniques have established deamidation as a key contributor to aging [9,10] and age-related diseases including cataracts [11], Alzheimer’s [12], Parkinson’s [13], cancer [14], and other degenerative diseases [15] as well as its relevance in biopharmaceutical development [16,17] and food science [18]. The biological and societal outreach of protein deamidation is depicted in Figure 1. As a driver of aging and age-associated disorders, understanding the mechanisms and consequences of deamidation may thus improve the diagnosis, treatment, and prevention of numerous pathologies.

The impact of deamidation can be illustrated by considering a typical eukaryotic cell with approximately 10,000 distinct proteins, each averaging 400 amino acid residues in length. Based on data from the Biological Magnetic Resonance Data Bank, which reports the occurrence of Asn (4.3%) and Gln (4.2%) residues, this translates to an estimated 400,000 potential deamidation sites across the proteome [19]. However, not all Asn or Gln residues undergo deamidation at the same rate. The typical half-lives for deamidation vary, ranging from 1 to 500 days for Asn and 100 to 5000 days for Gln at physiological pH and temperature. Robinson and Robinson [5] proposed that the surrounding amino acid sequences significantly influence the deamidation rates, positioning deamidation as an internal clock that regulates biological events. The rate of Asn deamidation has been shown to depend on the protein’s primary sequence, secondary and tertiary structures, and the surrounding cellular environment [6]. Factors such as pH, temperature, ionic strength, and the local protein microenvironment profoundly influence the reaction rates [5,6]. Given the extensive number of deamidation sites across a proteome, with shelf lives ranging from days to years, and the various factors affecting the rate of deamidation, it is essential to understand residue-specific and site-specific deamidation and its impact on protein structure, function, and its role in disease mechanisms and other domains.

Deamidation also poses major challenges for therapeutic protein development. Chemical degradation and modifications like deamidation, oxidation, and disulfide bond disruption affect protein stability, bioactivity, and immunogenicity [20]. Therapeutic proteins, peptides, and antibodies can undergo deamidation during production, storage, or even before administration to patients, leading to significant changes in drug purity, stability, bioactivity, and antigenicity [21,22]. Consequently, significant efforts have been dedicated to optimizing the manufacturing and storage conditions to minimize deamidation. Beyond biomedicine, deamidation has practical applications in food science and biotechnology. Deamidation enhances the functional properties of food proteins, such as emulsification, gelling, and stability [23], and contributes to the bioactivity of protein–saccharide conjugates with therapeutic potential such as antibacterial, antidiabetic, anti-osteoporosis, anti-inflammatory, anticancer, and immune-regulatory effects [24]. Additionally, deamidation is employed in quality control and historical protein analysis in art restoration, archeology, and forensics [25,26,27].

Deamidation-induced protein denaturation and aggregation have been implicated in numerous neurodegenerative and systemic diseases [1,28,29] and other pathologies like cataractogenesis [30,31], atherosclerosis [32], and diabetic secondary complications, further emphasizing the need to elucidate its mechanism. As both a driver of aging and a potential biomarker for disease, protein deamidation offers critical insights into human health and disease. Therefore, this review delves into recent developments in our understanding of protein deamidation, emphasizing its relevance across the biological and biopharmaceutical domains. Recognizing its impact on protein function and stability, further research is essential to harness deamidation as a diagnostic tool and therapeutic target in clinical and commercial settings.

## 2. Molecular Mechanisms of Protein Deamidation

The mechanism of protein deamidation has been extensively studied [33,34,35,36] and thus was not duplicated here, but is only briefly summarized. Deamidation of Asn primarily occurs at neutral or alkaline pH through the formation of a cyclic succinimide intermediate, which releases ammonia (NH_3_) upon formation. This intermediate can undergo hydrolysis either at α-carboxamide or β-carboxamide, yielding two products: aspartyl (Asp) and iso-aspartyl (isoAsp) residues [37]. The relative proportions of these products are governed by both kinetic and thermodynamic factors, with a typical hydrolysis ratio of approximately 1:3 for L-Asp to L-isoAsp in peptides containing Asp [38,39]. Additionally, the succinimide intermediate may racemize, forming D-enantiomers and subsequently yielding D-Asp and D-isoAsp in a similar 1:3 ratio upon hydrolysis. Under acidic conditions, Asn deamidates solely via direct side-chain hydrolysis, producing only Asp peptides [36]. At neutral or acidic pH, Asp isomerizes through dehydration, and Gln deamidates via a six-ring glutarimide intermediate [40]. The deamidation of Asn occurs significantly more frequently than that of Gln and follows a complex mechanism.

Although Asn and Gln residues are common in peptides and proteins, they do not always undergo age-dependent deamidation. Deamidation of Asn causes a 0.984 Da mass increase and added negative charge, detectable by high-resolution mass spectrometry. This seemingly minor change can result in significant alterations to the protein structure, stability, and function. In long-lived proteins, such as those in the brain, deamidation can accumulate over time, making it a key player in aging and neurodegeneration. Deamidation occurs both in vivo during development and aging, and in vitro, particularly during protein isolation or storage. However, extended incubation times during proteolysis, the use of alkaline buffers during protein digestion, and the reduction step with iodoacetamide, which is typically performed at 55–60 °C for 1 h, may contribute to deamidation. To mitigate these artifacts, modified workflows have been developed including an improved protocol that recommends tryptic digestion at pH 6 and deglycosylation at pH 5 for proteomes extracted with urea buffer [41]. Although proteogenomic approaches are widely used to assess protein abundance and map modification sites, bottom-up and shotgun proteomics inherently fall short in resolving the full complexity of the proteome. These methods may overlook PTMs, splice variants, and other proteoforms. To comprehensively characterize proteome diversity, future studies should incorporate top-down proteomics or complementary strategies capable of capturing intact protein species.

In addition to deamidation, nearly 300 other different PTMs have been identified and documented in the literature, broadly categorized into enzyme-mediated and nonenzymatic (spontaneous). Enzyme-mediated PTMs are further classified as reversible or irreversible based on their stability, whereas nonenzymatic PTMs occur spontaneously, often as a result of aging. Other contributing factors to spontaneous deamidation include oxidative stress and various environmental conditions such as pH, temperature, duration of exposure, protein structure and flexibility, ionic strength, and buffer composition. These elements collectively influence the chemical stability of susceptible residues, thereby affecting the rate and extent of deamidation. Protein L-isoaspartate O-methyltransferase (PIMT) has the potential to repair isoAsp residues. PIMT facilitates this process by converting isoAsp back to succinimide through the transfer of a methyl group from S-adenosyl-methionine (SAM) to the isoAsp side chain. This approach has been integrated into bioluminescence kits for rapid isoAsp detection [42,43]. However, while these kits provide convenience, their sensitivity and accuracy are inferior to those of mass spectrometric techniques. Thus, mass spectrometry remains the gold standard for PTM analysis due to its superior sensitivity and accuracy. The modifications, especially those that alter the charge or structure, are often low in abundance and difficult to detect amid unmodified peptides. Therefore, overcoming analytical challenges in identifying PTMs is critical, and various advanced mass spectrometric approaches (LC-MS/MS, ETD/ECD, and isotope labeling) have been developed to address this need [33,44,45]. Despite advances in understanding deamidation in vitro and in vivo, its precise role in aging and age-related diseases remains unclear.

## 3. Role of Deamidation in Protein Aging

Aging is a complex, multifactorial process marked by progressive biological, physiological, and neurological decline. It may result from programmed genetic mechanisms, as suggested by the longevity theory [46], or from cumulative cellular and molecular damage, as explained by theories like free radicals, wear and tear, and basal metabolism [47]. In the aging brain, the accumulation of protein abnormalities and inclusion bodies points to impaired proteostasis [48]. One hypothesis proposes that oxidative modifications render macromolecules resistant to lysosomal degradation [49]. This leads to the accumulation of non-degraded material, including lipofuscin, a key marker of aging in postmitotic cells [49]. Molecular aging largely arises from the exposure of biomolecules, particularly proteins, to reactive oxidants and nonenzymatic covalent modifications. Among the nonenzymatic modifications, deamidation has emerged as an indicator of molecular aging, a marker of molecular wear, and a functional modulator of proteins [1,50,51].

Protein aging is characterized by the gradual accumulation of spontaneous chemical modifications, among which deamidation is a major contributor. It alters gene expression, genomic instability, mutations, reduced cell division potential, cell death, impaired communication, tissue disorganization, organ dysfunction, and increased sensitivity to stress [52]. These effects correspond to the nine key hallmarks of aging including genomic instability, telomere attrition, epigenetic alterations, loss of proteostasis, mitochondrial dysfunction, deregulated nutrient-sensing, cellular senescence, stem cell exhaustion, and altered intercellular communication [53]. Oxidative stress caused by reactive oxygen species (ROS) leads to various PTMs, including carbonylation, DOPA formation, methionine and cysteine oxidation, and deamidation, ultimately altering the protein structure and function [54]. PTMs, particularly oxidation and deamidation, contribute to the age-related accumulation of dysfunctional proteins [55,56]. In summary, PTMs act as molecular switches regulating key cellular processes, including protein function, repair mechanisms, signaling, inflammation, genome stability, and epigenetic control. With age, PTM regulation becomes less precise, contributing to cellular dysfunction and organismal aging. Deamidation and its degree are crucial factors in the aging process.

Before 1970, nonenzymatic deamidation was mistakenly considered an experimental artifact, as most biochemical processes were known to be enzyme-driven. However, it was later shown that the deamidation of Asn plays a regulatory role in processes such as protein turnover (e.g., cytochrome c and aldolase) [57,58,59], tracking enzyme catalytic cycles in triosephosphate isomerase [60], and facilitating the time-dependent monitoring of DNA repair as well as the regulation of apoptosis in Bcl-XL [61]. These findings led to the hypothesis that deamidation acts as a molecular clock, with the extent of modification correlating with protein age. Unlike other modifications, deamidation occurs nonenzymatically at biologically relevant rates and can significantly alter the protein structure. Using experimentally verified computations, Robinson and Robinson [9] determined deamidation rates in 49 Drosophila proteins, demonstrating their role in regulating aging, development, and biochemical processes. Thus, nonenzymatic deamidation, occurring independently of enzymes and tunable over time spans from hours to years, serves as a genetically programmed, structural, and temporal regulator, a unique and effective molecular clock.

The human lens serves as an ideal model for studying the role of deamidation in aging due to the long-lived nature of crystallin proteins, [62], which do not turnover post-synthesis. The human lens is composed of nine major crystallin proteins (αA, αB, βA1/A3, βA4, βB1, βB2, γS, γC, and γD), comprising 150 Asn and Gln residues [63]. Although the prevalence of Asn/Gln residues in crystallins is not significantly different from the average for all proteins, deamidation increases with age and may influence the lens properties [64]. Studies have shown that replacing a single Asn with Asp in βB1-crystallin alters the physical properties, increasing aggregation tendency [65], while the deamidation of Gln in βA3, βB1, and γD-crystallins reduces protein stability [30,66]. Wilmarth et al. [62] reported that insoluble aggregates that formed from soluble crystallins in the lens exhibited higher levels of deamidation when comparing young (3-year-old) and aged (70-year-old) lenses. Their findings revealed that these aggregates show significantly higher deamidation levels in older lenses, indicating a role in age-related visual decline. Similar age-associated deamidation patterns have been observed in other long-lived proteins such as collagen [67] and histone [68,69] in aging rodents. The accumulation of deamidated proteins has been implicated in various age-related diseases. These modifications may also enhance immunogenicity, potentially triggering autoimmune responses against self-proteins with altered epitopes.

### 3.1. Deamidation and Neurodegenerative Diseases

**Figure 2:**
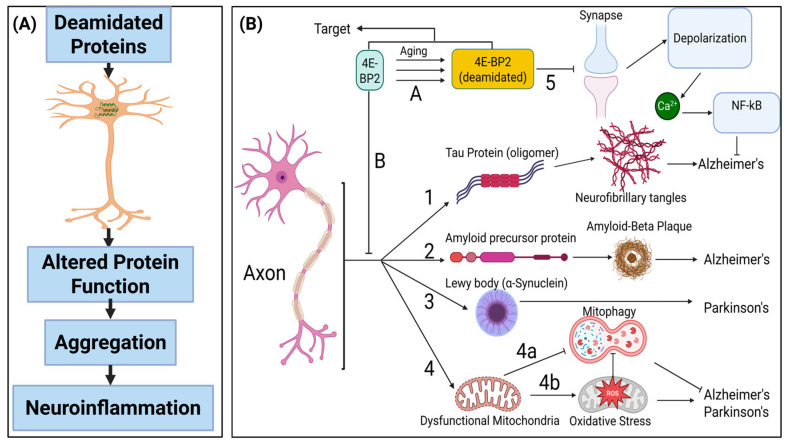
Schematic representation of the deamidation-induced neurodegenerative cascade. (**A**) Deamidation induces protein misfolding and aggregation, evading proteostasis and triggering microglial activation, which drives neuroinflammation and neuronal dysfunction. (**B**) The unified theory of neurodegeneration pathogenesis (adopted from Davis Joseph [70]). Axon deamidation, establishes the link between the four fields of deamidation (Step A), translational control (Step B), neurodegenerative diseases (Options 1 to 4), and oxidative stress (Option 4).

Neurons are the fundamental units of the nervous system, forming the structural and functional basis of the brain and spinal cord. As essential and largely non-renewable cells, any damage or loss of neurons is typically irreversible. A hallmark shared by many neurodegenerative diseases is the misfolding and aggregation of proteins, which initiates a cascade of events leading to progressive neuronal dysfunction and degeneration, a process collectively referred to as neurodegeneration. This process of neurodegeneration is central to disorders such as Alzheimer’s disease (AD), Parkinson’s disease (PD), Huntington’s disease (HD), and amyotrophic lateral sclerosis (ALS). PTMs such as deamidation promote protein aging by gradually altering conformation, increasing the aggregation propensity, and enabling escape from cellular quality control, thereby contributing to neurodegenerative pathology. The schematics of the consequences of deamidation in neurons are shown in Figure 2A. A unified theory based on axonal deamidation was proposed by Davis Joseph [70] (Figure 2B), highlighting the role of 4E-BP2 deamidation in neuronal projections, primarily axons, in regulating neurodegenerative diseases such as Alzheimer’s and Parkinson’s. This mechanism integrates four key biochemical pathways: deamidation, translational control, neurodegeneration, and oxidative stress.

Proteins depend on accurate three-dimensional folding to preserve their function. When they misfold, they can clump together into fibrils and inclusions, interfering with normal cellular activities. These abnormal protein accumulations contribute to oxidative stress, cytoskeletal disruption, organelle dysfunction, apoptosis, and other harmful cascades [71]. For example, in AD, amyloid-beta aggregates form plaques that impair neuronal communication, while tau proteins form tangles that interfere with axonal transport. PD involves the aggregation of α-synuclein into Lewy bodies, damaging dopamine-producing neurons, and HD is characterized by huntingtin protein aggregates that cause widespread neuronal damage. These aggregates not only disrupt cellular function, but also trigger inflammatory and stress responses, exacerbating neuronal damage. Overall, protein misfolding and aggregation are central to the pathology of over 40 neurodegenerative disorders, highlighting the critical role of protein structure in maintaining neuronal health. Protein misfolding and aggregation caused by deamidation, along with their associated diseases, are summarized in Table 1.

In the absence of clinically diagnosed neurological diseases, autopsy studies of aged brains exhibit hallmark pathological features such as amyloid plaques, neurofibrillary tangles, Lewy bodies, synaptic degeneration, neuronal loss, and reduced brain volume [49]. These features vary significantly across individuals and brain regions, and their precise contribution to neurodegeneration remains uncertain, possibly due to their limited extent or subtle impact. Age-related oxidative damage, observed across tissues and species, exacerbates this vulnerability. While high levels of oxidative stress are known to cause cellular damage and inflammation, lower levels may paradoxically trigger adaptive protective mechanisms. PTMs, particularly deamidation, can contribute to cellular damage that exceeds the threshold of adaptive protective mechanisms, thereby playing a critical role in the development of pathological hallmarks. The causal relationship between oxidative stress and aging, however, is still under active investigation. In addition, growing evidence highlights the profound influence of lifestyle, diet, environmental toxins, and substance use on health span, longevity, and the risk of developing neurodegenerative diseases [96]. However, the underlying molecular pathways through which these factors exert their effects remain largely elusive. Hence, elucidating the role of deamidation, in promoting or preventing protein misfolding, could uncover novel therapeutic targets for halting or reversing the progression of neurodegenerative diseases.

**Figure 3:**
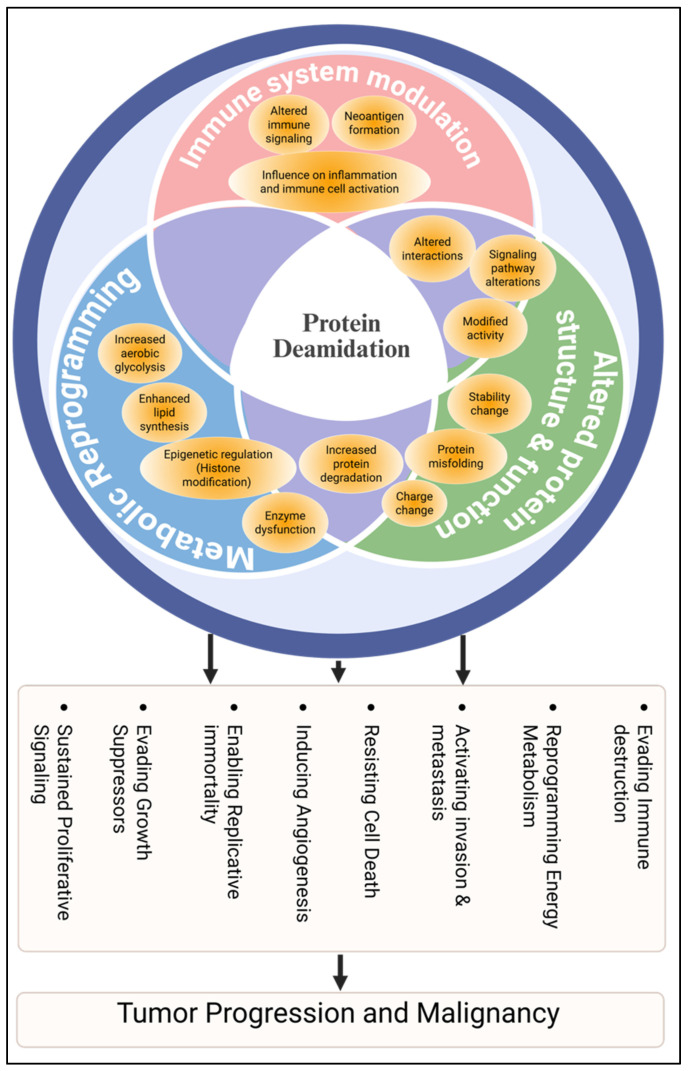
Impact of protein deamidation on various cellular processes and the proposed mechanism of tumor progression.

Dementia is a progressive neurodegenerative disorder that affects over 50 million people worldwide, with prevalence increasing with age. According to the World Alzheimer Report, the number of people living with dementia is projected to reach approximately 82 million by 2030 (Figure 3). Genomics has revealed that genetic variations play a significant role in the onset, progression, and susceptibility of dementia. Familial Alzheimer’s disease (AD) arises from mutations in APP, PSEN1, and PSEN2, which lead to abnormal amyloid-beta production and plaque formation [97]. The APOE-ε4 allele is a major genetic risk factor for sporadic AD [98]. Frontotemporal dementia (FTD) has been linked to mutations in MAPT and GRN, while vascular dementia has been linked to NOTCH3 polymorphisms that compromise vascular integrity [99,100]. Additionally, variants in TREM2, CLU, CR1, and BIN1 influence immune response, lipid metabolism, and amyloid clearance, thereby increasing the risk of dementia [101]. While genomics provides a foundational blueprint of DNA sequences, proteomics offers insights into the functional molecular machinery driving cellular processes and disease. A single gene can produce multiple protein isoforms through alternative splicing and PTMs, which impact disease mechanisms but are mostly undetectable by genomic studies. PTMs can profoundly affect protein function, especially under pathological conditions, and unlike the static genome, protein abundance is dynamic and responsive to environmental influences. Neurodegenerative diseases often exhibit distinct protein abundance profiles, and modified proteins may serve as biomarkers for diagnosis and prognosis. Given that most drugs target proteins, proteomics plays a vital role in elucidating disease mechanisms and guiding therapeutic development. In summary, while genomics provides critical insights into genetic predispositions, proteomics offers a real-time, functional perspective on disease pathology. Integrating multi-omics approaches, including genomics, transcriptomics, proteomics, and metabolomics, through advanced bioinformatics tools holds great promise for deepening our understanding of dementia and shaping future therapeutic strategies.

Proteomics enables high-throughput biomarker screening, facilitating early disease diagnosis, therapeutic development, and the investigation of disease mechanisms. For example, the proteomic analysis of postmortem amyloid plaques identified over 400 distinct proteins [102]. Advanced techniques, such as ultracentrifugation-electrostatic repulsion hydrophilic interaction chromatography (UC-ERLIC) combined with mass spectrometry, have enabled detailed profiling of protein aggregates in dementia-affected brain tissue. Using this approach, Adav et al. [29] found significant enrichment and deamidation of proteins such as S100A9, ferritin, hemoglobin subunits, creatine kinase, and collagen within amyloid plaques. Notably, structural analysis revealed exclusive deamidation of S100A9’s calcium-binding motifs in aggregated fractions, altering its charge and potentially promoting pathological protein aggregation. However, the study’s limited sample size constrains its generalizability, underscoring the need for further validation. Myelin basic protein (MBP), essential for myelination and cellular signaling in the brain [103] is susceptible to post-translational modifications like deamidation and citrullination [104]. Gallart-Palau et al. [105] reported hyper-deamidation at Gln-82 in MBP predominantly in female dementia patients, impairing degradation and leading to protein accumulation in the temporal lobe. These findings highlight sex-specific molecular differences in AD + CVD neuropathology and suggest avenues for gender-targeted interventions.

Ceruloplasmin (Cp) is a multicopper ferroxidase primarily produced by hepatocytes and released into the blood [106]. In the CNS, it is expressed by astrocytes as a GPI-anchored isoform and secreted into the cerebrospinal fluid by choroid plexus epithelial cells [107]. Cp has two asparagine-glycine-arginine (NGR)-motifs: the first (568NGR) is surface-exposed, and the second (962NGR) is buried. The 568NGR motif deamidates at 37 °C and basic pH, conditions that accelerate Asn aging [108], while the 962NGR motif deamidates only under particular conditions. Cp in the cerebrospinal fluid (CSF) of PD patients was found to be more oxidized and deamidated than in healthy subjects [78], with deamidation predominantly at the hidden 962NGR. Zanardi and Alessio [79] suggested that brain protein deamidation may lead not only to loss of function, but also to a toxic gain of function.

The accumulation of deamidated residues in long-lived proteins can lead to aberrant signaling and has been implicated in age-related and neurodegenerative diseases. Deamidated Cp binds to integrins and triggers intracellular signaling on choroid plexus epithelial cells, changing cell functioning [79]. The Na^+^/K^+^-ATPase functions not only as an ion transporter, but also plays a critical role in various signal transduction pathways, many of which are regulated through protein–protein interactions involving the pump itself. Adav et al. [28] reported significant dysregulation of the sodium-potassium transporting Na^+^/K^+^-ATPase, accompanied by an upregulation of PIMT. Their findings highlighted deamidation of the ATP1A1 and ATP1A2 subunits of the Na^+^/K^+^-ATPase, along with impaired isoaspartyl residue repair by PIMT. The authors proposed that such deamidation may interfere with electrostatic interactions crucial for the E1 phosphorylation state, potentially impairing both ion transport and associated signaling functions. In addition to spontaneous deamidation, enzymatic deamidation also plays a critical role in regulating the signal transduction involved in fundamental biological processes such as innate immune responses [109]. In signaling cascades, such as those involved in immune responses, cell growth, and apoptosis, deamidation can modulate the activity of key signaling proteins and transcription factors. For instance, the deamidation of components in kinase pathways or membrane-bound receptors may impair phosphorylation events or disrupt scaffold protein assembly.

### 3.2. Deamidated Proteins as a Biomarker

Advances in health care and social support have extended the life span and improved the quality of life for elderly individuals with neurodegenerative diseases. Despite improved care, neurodegenerative diseases remain incurable due to their complex, multifactorial causes. It is essential to develop new biomarkers to enhance our understanding of each disease’s etiology and improve screening and diagnosis, enabling timely clinical intervention. The diagnosis of neurodegenerative diseases typically relies on clinical symptoms, such as cognitive decline and movement disorders, alongside neuroimaging techniques like positron emission tomography (PET) and magnetic resonance imaging (MRI) or computerized tomography [110]. In recent years, molecular biomarkers reflecting protein pathology and aggregation have garnered increasing attention in neurodegenerative disease research. While CSF biomarkers like amyloid β (Aβ), tau, and α-synuclein offer valuable insights, CSF collection is invasive (lumbar puncture), complex, and expensive. Building on these findings, multiplex biomarker strategies have been proposed to enhance diagnostic accuracy by integrating multiple biomarkers, enabling differentiation between distinct disease states or the simultaneous classification of coexisting conditions [111]. This has led to growing interest in blood-based biomarkers. Ray et al. [112] analyzed 259 plasma samples from individuals ranging from presymptomatic to late-stage Alzheimer’s and various controls, measuring the levels of 120 known signaling proteins using sandwich ELISAs. They identified eighteen proteins that distinguished between the blinded Alzheimer’s and control samples with ~90% accuracy and predicted the progression from mild cognitive impairment to Alzheimer’s within 2–6 years. Immunology-based studies often target proteins linked to AD progression such as low or ultra-low-abundance cytokines in the blood. Low-abundance proteins are challenging to detect by mass spectrometry, and when detected, their low signal levels result in poor accuracy and high variability. However, Yang et al. [113] focused on high abundant putative protein biomarkers including complement factor I, ceruloplasmin, plasma protease C1 inhibitor, alpha-2-macroglobulin, and fibrinogen in AD blood. In addition to these, neurofilament light protein (NfL) [114], apolipoprotein E (APOE), phosphorylated tau (p-tau), and glial fibrillary acidic protein (GFAP) [115] have also been proposed as plasma biomarkers for AD.

A promising approach by Wang et al. [116] suggests that impaired isoAsp repair leads to deamidation buildup before AD onset, making deamidation-based biomarkers highly sensitive for early detection. In a study of 140 participants, including individuals with AD, mild cognitive impairment (MCI), vascular dementia (VaD), frontotemporal dementia (FTD), PD, and healthy controls, they found significantly elevated isoAsp levels in MCI, FTD, and VaD, and reduced anti-aHSA IgG levels in MCI and VaD. Deamidated blood albumin outperformed traditional biomarkers (Aβ42/Aβ40, NfL, GFAP, and p-tau181), achieving up to 92% accuracy in distinguishing MCI from healthy controls. Further supporting and validating this, a 2023 Amsterdam cohort study by Wang et al. [76] reported a strong correlation between the isoAsp levels in human serum albumin (HSA) and AD. Deamidation products were notably elevated in VaD and FTD, less in PD, and consistently correlated with cognitive decline [76,116]. Collectively, the isoAsp levels, anti-aHSA IgGs, and IsoAsp/IgG ratio show strong potential as early biomarkers of neurodegeneration.

## 4. Protein Deamidation in Cancer

Spontaneous deamidation is considered a key factor in protein aging due to three main reasons: (1) deamidation promotes protein degradation [117]; (2) deamidated proteins accumulate with age; and (3) the repair system involving PIMT declines over time [118], allowing IsoAsp residues to build up. Acting as a molecular clock, deamidation often leads to a decrease in protein activity. For instance, it impairs calmodulin’s ability to activate Ca^2+^/calmodulin-dependent kinase [119], reduces muscle aldolase activity by 50% in aged mice, and disrupts substrate binding and catalysis in human triosephosphate isomerase [120]. Thus, deamidation represents an irreversible alteration of the information originally encoded in the genome and translated during protein synthesis, changing the protein’s primary structure, introducing a negative charge, and often impacting its secondary and tertiary structures. Deamidation can be triggered in living cells as part of the DNA damage response, altering Bcl-xL’s survival function and becoming a key mechanism for regulating apoptosis [19].

In cancer, where cells exhibit increased metabolic demands and rely heavily on glycolysis [121], protein deamidation has gained attention as a potential therapeutic target. Triosephosphate isomerase (TIM) is a key glycolytic enzyme essential for the interconversion of glyceraldehyde-3-phosphate and dihydroxyacetone phosphate. Due to the reliance of cancer cells on glycolysis, a deamidated form of human TIM (HsTIM) has been proposed as a potential target for cancer therapy [122]. In breast cancer cells, a deamidated form of human TIM (HsTIM) showed heightened sensitivity to thiol-reactive drugs like rabeprazole and auranofin. Both drugs inhibited HsTIM enzyme activity and induced selective cell death [14]. Similarly, deamidation can shift the function of RelA from activating NF-κB target genes to increasing the abundance of glycolytic enzymes, redirecting the cell’s response from inflammation to aerobic glycolysis [123].

Aberrant deamidation impacts key regulatory proteins, such as transcription factors and signaling mediators, affecting their stability, interactions, and cellular localization. The Bcl-2 protein family—which includes both pro- and anti-apoptotic members such as BAX, BAK, Bcl-2, and Bcl-xL—regulates mitochondrial apoptosis. Deamidation of Bcl-xL compromises its antiapoptotic function, disrupting the apoptotic balance and enhancing tumor cell survival. Bcl-2(B-cell lymphoma-2) family proteins, which include proapoptotic (e.g., BAX, BAK), antiapoptotic (e.g., Bcl-2, Bcl-xL), and BH3-only proteins (e.g., BIM, BID, PUMA), regulate the mitochondrial pathway of apoptosis by interacting with the mitochondrial outer membrane [124]. Deamidation of Bcl-xL and other apoptosis-related proteins compromises its antiapoptotic function, disrupting the apoptotic balance and enhancing tumor cell survival [61,125]. In many cancers, this balance is disturbed through the overexpression of antiapoptotic Bcl-2 family proteins or protein deamidation. These alterations have been implicated in various malignancies including chronic myeloid leukemia (CML), pancreatic, ovarian, and small-cell lung cancers [126,127].

In addition to modulating cell death pathways, deamidation influences signaling pathways, protein–protein interactions, and tumor immune evasion, highlighting its potential role in tumor progression and as a therapeutic target. For example, apelin-induced glutamine amidotransferase activity leads to HMGA1 deamidation, promoting lipid synthesis and tumor growth in non-small cell lung cancer [128]. Deamidation of the Bcl-XL protein inhibits its antiapoptotic ability and leads to apoptosis induced by alkylating agents in Rb-deficient tumor cells [129]. The role of protein deamidation on innate immune signaling pathways, which can influence tumor immunity and cancer progression has been reviewed by Zhao et al. [109]. The influence of protein deamidation and possible mechanisms is presented in Figure 3. The amino acid Asn itself, while not directly degraded in mammals, plays a vital role in translation, senescence regulation, and cancer metabolism [130]. Through the mTORC1 signaling pathway, it supports the uptake and utilization of other intracellular nutrients, helping meet the energy demands of rapidly proliferating cancer cells. Asn also influences metastasis by regulating epithelial–mesenchymal transition (EMT). Its role and metabolic fate have been explored across various cancers including leukemia, breast cancer, melanoma, lung cancer, and colorectal cancer [131].

## 5. Protein Deamidation in Forensics and Archeology

The deamidation of Asn and Gln has long been studied in protein aging and degradation. These modifications persist across forensic, archeological, and paleontological contexts, providing valuable insights into sample chronology and preservation conditions. While paleontological materials typically include bones or teeth, archeological samples encompass a broader range of organic traces such as food residues in ceramics, dental calculus, soft tissues (e.g., skin, wool), and paint binders. In forensic science, human skeletal remains are central to reconstructing past demographics, epidemiological patterns, and individual identification. Estimating the post-mortem interval (PMI) remains challenging, especially during advanced stages of decomposition, due to the complex interplay of physical, biological, and chemical changes the body undergoes. The interplay of endogenous factors (e.g., body size, age, health) and exogenous variables (e.g., temperature, humidity, soil composition, burial conditions, scavenger activity), with added complexity in aquatic environments, make it more complex [132]. The challenge is further amplified for bodies found in water. In such cases, bone proteomics remains a novel approach for PMI estimation. Recent studies have focused on Asn deamidation, protein abundance, and marker protein degradation [133,134]. For instance, Procopio et al. [133] identified a decline in plasma and muscle proteins with increasing PMI, alongside significant increases in biglycan deamidation, an important protein for bone growth and mineralization [135]. Among the five deamidated proteins analyzed, only biglycan showed a statistically significant correlation with PMI, suggesting its potential as a biomarker for estimating PMI between one and six months.

Collagen, the most abundant structural protein in mammals and predominant in connective tissues like bone cartilage, tendons, ligaments, and skin, has demonstrated extraordinary longevity [136]. Studies have recovered collagen from archeological specimens nearly one million years old [137], with its alpha-1(I) and alpha-2(I) chains being especially durable. Additionally, biglycan, and to a lesser extent, chondroadherin, correlated positively with geological age and deamidation extent. A recent study found that deamidation levels in collagen peptides from a 700,000-year-old horse specimen preserved in permafrost were lower than those in 11,000–19,000-year-old mammoth specimens from temperate regions [138]. This finding underscores the significant influence of storage conditions on protein preservation and highlights the relevance of thermal age over chronological age in molecular degradation. The deamidation of collagen in bones from similar environments supports the concept of “thermal age”—a measure of chronological age adjusted for environmental temperature exposure [139,140]. Other than temperature, environmental conditions, like pH, humidity, light exposure, and protein structure, greatly influence the deamidation rates. Because the storage conditions in museums, archeological samples, and buried remains can vary and are often uncertain over centuries, this variability complicates direct comparisons of the deamidation levels across samples and even across different archeological sites. However, adjusting the chronological ages of samples according to their thermal ages enables more meaningful cross-regional analyses [140]. This methodology has also been applied in studies of ancient textiles.

The protein keratin, abundant in hair and wool, shows promise in both forensic and archeological research due to its environmental resistance. The hair shaft is a primary source of biological evidence commonly found and collected at crime scenes. Hair proteomics, aided by mass spectrometry, now enables individual identification via genetically variant peptides (GVPs) [141,142]. Adav et al. [143] explored the possibility of utilizing hair proteomics phenotyping in forensic science to differentiate individuals across various ethnic groups, sex and age. Furthermore, hair protein deamidation has been explored [144,145] but is limited to detecting the site of modifications alone. Araki and Moini [146] demonstrated that deamidation in sheep wool correlates with age (up to ~400 years), highlighting its potential as a biomarker, while Solazzo et al. [147] used α-keratin markers with 2–5 deamidation sites from sheep wool peptide profiles to evaluate the extent of deamidation in aged, buried, and archeological samples. Their findings indicate that deamidation rates are influenced by both the protein structure and environmental factors. While studies on aged wool have confirmed the time-dependent nature of deamidation under constant temperature, they highlighted the necessity for further research into the effects of environmental conditions (such as light and humidity) and wool treatments (including dyeing, mordanting, and metal wrapping). Scalp hair, primarily composed of keratin, exhibits exceptional biochemical stability, rendering it highly resistant to decomposition and environmental degradation. Consequently, the analysis of keratin deamidation in hair presents a valuable tool for forensic investigations. However, external factors such as cosmetic hair treatments and ultraviolet (UV) radiation exposure must be critically evaluated, as they may significantly influence the extent and interpretation of deamidation patterns

Deamidation has proven to be a valuable tool in identifying protein residues in archeological ceramics. Chowdhury and Buckley [139] analyzed proteins from Neolithic-era ceramics at Çatalhöyük [148] to study deamidation. They identified a range of food proteins including β-lactoglobulin, caseins (α, β, κ), hemoglobin (mainly Cervinae), legumins (Pisum sativum, Fabae, Vicia), hordeins (Hordeum vulgare), α-1-purothionin (Triticae), and serpins (Triticae, Hordeum vulgare). All proteins in the samples exhibited similar deamidation levels, likely due to their shared chronological period and site. Notably, β-lactoglobulin displayed higher deamidation in the ceramics compared with dental calculus, which the authors attributed to the ceramics’ greater age and the warmer climate in which they were found [139]. The protein amelogenin, which is encoded by the sex-linked AMELX and AMELY genes, allows for sex estimation through proteomic analysis in both modern and archeological samples [149]. In all modern and archeological samples tested, the authors consistently found significant deamidation in both identified peptides and those spanning the dimorphic sequence. This consistent pattern indicates that deamidation is a reliable marker of endogeneity. It can be used not only in archeological bone, but also in distinguishing endogenous proteins from contaminants in ceramics, as demonstrated in a study on Endomesolithic ceramics from Friesack 4, Germany [150].

Proteomic strategies have expanded beyond the forensic and archeological sciences into the analysis of historic artworks. Proteinaceous binders in paintings, such as casein and collagen, deteriorate over time [151]. Proteomic methods, as highlighted by Leo et al. [27], serve as an effective complement to traditional amino acid composition analysis, offering deeper insights into the aging and degradation of protein-based materials in art. The analysis of samples from the Camposanto Monumentale frescoes revealed protein deterioration, including decreased levels of Met, Lys, and Tyr, alongside the presence of amino malonic acid, which results from the oxidation of Ser, Phe, and Cys [26]. The deamidation of Asn and Gln has been identified as a key marker of protein aging in these materials [26,151]. These discoveries underscore the importance of molecular signatures in characterizing protein-containing artworks and historical artifacts, while also highlighting the potential of proteomic and deamidation approaches for studying the molecular aging effects. Asn deamidates at a rate ten times faster than Gln, leading researchers to propose that measuring Gln deamidation could serve as a reliable method for assessing the aging of ancient artifacts. Notable proteins studied in this context include osteocalcin, collagen, and keratin in bones and textiles, while casein and collagen are crucial for analyzing binders in old paintings [25,139].

## 6. Protein Deamidation in Biopharmaceuticals and Drug Development

Monoclonal antibodies (mAbs) are a key and rapidly growing class of biological drugs used to treat cancer, autoimmune disorders, infectious diseases, and organ transplantation [152]. Over the past 30 years, the FDA has approved more than 70 full-length mAbs and related fragments, with over 50 more in late-stage development [153]. These immunoglobulins (Ig) fall into five isotypes such as IgG, IgM, IgA, IgE, and IgD with multiple subtypes. Despite carefully optimized formulation and rigorous process development, these immunoglobulins, remain susceptible to spontaneous degradation during manufacturing, storage, handling, and clinical use. Key PTMs, such as methionine or tryptophan oxidation, carbonylation and especially deamidation, can alter protein conformation, compromising both chemical and physical stability [154]. Asn deamidation and Asp isomerization are among the most prevalent covalent modifications contributing to charge heterogeneity in monoclonal antibodies (mAbs) [155]. These modifications can affect the structural integrity, biological activity, and pharmacokinetics of mAbs, potentially compromising their therapeutic efficacy and safety. Moreover, the resulting charge variants pose analytical challenges, necessitating robust characterization methods such as ion exchange chromatography and mass spectrometry to ensure product consistency and regulatory compliance.

Deamidation can compromise both the binding affinity and biological function. For example, a therapeutic IgG1 exhibited over a 50% loss in antibody-dependent cell-mediated cytotoxicity (ADCC) after four months of heat stress at 40 °C, attributed to deamidation at residue N325 [16]. Furthermore, upstream process parameters, particularly temperature and pH, have been shown to significantly influence the formation of acidic charge variants. Dengl et al. [156] studied several IgGs produced in Chinese hamster ovary (CHO) cells and reported that standard culture conditions (37 °C, neutral pH) led to approximately 20% deamidation in one mAb including a non-critical site within the complementarity-determining region (CDR). Notably, in vitro deamidation rates closely mirrored the in vivo rates due to similar environmental conditions. Similarly, Kaneko et al. [157] observed increased deamidation and charge heterogeneity during CHO cell culture, further supporting the impact of upstream factors on product quality. Given these findings, charge variant analysis plays a critical role in ensuring lot-to-lot consistency and detecting product instabilities throughout mAb production [158]. As a critical quality attribute, deamidation can increase the risk of aggregation and lead to functional and pharmacological changes such as reduced therapeutic efficacy, altered immunogenicity, impaired target recognition, and changes in vivo half-life [159,160,161]. Therefore, the relationship between mAb deamidation and upstream processing conditions should be a central focus in biologics development.

Biomanufactured therapeutic proteins, including peptides, vaccines, monoclonal antibodies (mAbs), and viral capsids, experience both physical and chemical stress during upstream process, leading to PTM accumulation [17,162]. For example, Giles et al. [17] showed that spontaneous deamidation significantly impaired adeno-associated virus 8 (AAV8) transduction by triggering early vector activity loss to rapid deamidation at specific asparagine residues. By introducing mutations to stabilize side-chain amides, they improved the vector performance and reduced batch variability, which remains an essential consideration in biologics manufacturing. Therefore, to evaluate and mitigate potential risks, clinical-stage mAbs undergo stress testing, such as exposure to heat and extreme pH conditions, to accelerate degradation processes that may occur during manufacturing and storage. Stress tests and related studies conducted during lead selection are crucial for identifying stable candidates, detecting degradation-prone sites and informing sequence re-engineering. The insights gained from these evaluations contribute to improved product stability, more efficient development, and greater manufacturing consistency. However, unintended or uncontrolled PTMs during production and storage can compromise the efficacy, potency, and overall stability. Therefore, the identification and characterization of proteins susceptible to Asn/Asp deamidation and Asp isomerization, along with their degradation variants, are vital steps in the development of stable and effective therapeutics.

The degradation half-life of Asn and Asp residues in small peptides can vary dramatically depending on the adjacent C-terminal (n + 1) residue, as shown by Robinson and Rudd [64]. Using pentapeptides, Robinson et al. [163] further demonstrated that Asn was more prone to chemical degradation when followed by Gly, Ala, Ser, or Thr. While these findings offer insights, peptide-based observations may not fully apply to folded proteins. For instance, Lu et al. [158] observed significant variability in deamidation and isomerization across 131 clinical-stage antibodies, independent of the peptide sequence patterns. Nevertheless, in short therapeutic peptides, sequence motifs remain important for design and engineering. Deamidation also impacts monoclonal antibody (mAb) stability in a pH-dependent manner, having a minimal effect at neutral pH but potentially affecting colloidal stability and promoting aggregation under acidic conditions [164]. Pace et al. [165] found that deamidation rates in IgG1 increased as the pH rose from 5.3 to 8.3. Additional factors such as formulation pH, solution viscosity, polarity, stabilizers, excipients, metal ions, protein structure, temperature, buffer strength, and sequence motifs also influence deamidation [21]. A thorough understanding of these parameters enables the early prediction and control of deamidation during development, ultimately enhancing therapeutic stability, efficacy, and safety.

Predicting deamidation and identifying modification sites is crucial for drug development. However, traditional approaches, such as long-term storage studies, are time-consuming, and accelerated stability tests often fail to accurately represent deamidation under real storage conditions. To address these limitations, computational and machine learning models have been developed to predict deamidation and isomerization. Several studies have introduced in silico tools that estimate the deamidation sites and rates based on known protein 3D structures [166,167]. Sydow et al. [168] analyzed the structural properties of Asn and Asp residues in antibody variable domains, developing machine learning models to predict the degradation propensity within the CDRs of mAbs. Yan et al. [169] constructed a decision tree model using five IgG1 and IgG4 mAbs to predict the Asn deamidation propensity based on structural parameters, facilitating the early identification of degradation hotspots. A physics-based computational model was also proposed to identify degradation sites by evaluating free-energy barriers along the pre-chemical conformational step and chemical reaction pathway, using classical and quantum mechanics/molecular mechanics as well as molecular dynamics simulations [170]. More recently, Irudayanathan et al. [171] used proton-affinity calculations with semi-empirical quantum mechanics and molecular dynamics simulations to study Asn and Asp degradation in 131 therapeutic antibodies, identifying secondary structure, side-chain conformation, and solvent accessibility as key factors in isomerization and deamidation.

Bults et al. [172] investigated the in vivo deamidation of the therapeutic monoclonal antibodies trastuzumab and pertuzumab, both of which are widely used in the treatment of HER2-positive breast cancer. Their study focused on quantifying deamidation at critical Asn55 in the heavy chain (HC) of trastuzumab and Asn54 in the HC of pertuzumab, which are essential for HER2 receptor binding. They found a clear correlation between the extent of deamidation at these sites and reduced HER2 binding affinity, ultimately leading to diminished biological activity. Notably, fully deamidated trastuzumab lost its tumor-inhibitory function, highlighting the significant impact of this structural modification on therapeutic efficacy. Spanov et al. [173] systematically analyzed the charge heterogeneity and deamidation susceptibility of pertuzumab. Pertuzumab is used in combination with either trastuzumab or ado-trastuzumab emtansine for the neoadjuvant treatment of metastatic HER2-positive breast cancer [174].

## 7. Future Perspective

Profiling the proteome is complex and challenging due to various technical limitations. Proteome profiling by LC-MS/MS is inherently complex and is constrained by multiple technical limitations that affect the sensitivity, reproducibility, and proteome coverage. Sample preparation introduces biases due to protein solubility issues, incomplete digestion, and peptide losses. The limited dynamic range of mass spectrometers and variable ionization efficiencies hinder the detection of low-abundance proteins. Data-dependent acquisition (DDA) suffers from stochastic sampling and missing values, while data-independent acquisition (DIA) requires computationally intensive deconvolution and spectral libraries. Instrumental limitations, including scan speed and mass resolution, further restrict peptide identification and quantification. Quantitative approaches face challenges such as ratio compression in isobaric labeling and variability in label-free methods. Bioinformatic analysis is complicated by database dependency, difficulty in localizing PTMs, and algorithmic inconsistencies. Additionally, low throughput, high operational costs, and limited reproducibility remain significant barriers to large-scale or clinical implementation.

However, advances in mass spectrometry and bioinformatics are deepening our understanding of protein deamidation and revealing its broad implications in cellular biology and clinical science. The deamidation of Asn and Gln residues alters the protein structure, function, and stability, influencing a range of physiological and pathological processes. Future investigations are expected to elucidate the mechanistic pathways governing spontaneous and enzymatic deamidation, with particular emphasis on its regulation by cellular stress responses, redox homeostasis, metabolic flux, and proteostasis networks. The development of high-resolution mass spectrometric techniques, isotope labeling techniques, computational modeling, and site-specific deamidation assays will enable unprecedented resolution in the detection, localization, and quantification of deamidation events in complex biological matrices. Advances in proteomic profiling and structural biology will enhance our ability to map age-dependent deamidation events, offering new perspectives on cellular aging and potential anti-aging interventions. Moreover, the integration of structural bioinformatics, molecular dynamics simulations, and machine learning models will facilitate the prediction of deamidation-prone motifs and their functional consequences. Given the emerging roles of deamidation in aging, neurodegeneration, oncogenesis, immunogenicity, and protein therapeutics, future interdisciplinary research is well-positioned to uncover novel biomarkers, therapeutic targets, and quality control parameters across both fundamental and translational domains of biomedical science.

The accumulation of deamidated proteins in neurological tissues is increasingly implicated in the pathogenesis of disorders such as AD, PD, and HD. The current literature suggests that deamidation is a driver of protein misfolding and aggregation. However, integrative studies combining genomics, proteomics, and neuroimaging may pave the way for identifying deamidation-based biomarkers and therapeutic targets for neurodegeneration. Beyond neurodegeneration, deamidation holds promise as a diagnostic and prognostic biomarker across various diseases, though standardized detection and clinical validation are crucial for clinical translation. In cancer, aberrant deamidation can modulate oncogenic signaling, immune evasion, and therapy resistance as well as modulate the function of the protein. Future studies targeting deamidation-associated pathways could support the development of personalized diagnostics and therapeutics.

Protein deamidation is a dynamic, context-dependent modification with broad biological and translational relevance. Its roles span from influencing neurodegeneration and cancer progression to serving as a molecular clock in forensic and archeological studies. Future prospects in these fields include improving the quantification techniques and environmental corrections to improve the accuracy of age estimation in both forensic and archeological contexts. In biopharmaceuticals, deamidation is a critical quality attribute influencing the stability, efficacy, and immunogenicity of therapeutic proteins. In silico tools and machine learning can improve the prediction of deamidation-prone sites, while protein engineering strategies, such as site-directed mutagenesis and sequence optimization, offer ways to enhance resistance without compromising function. Combined with optimized formulations (e.g., pH, buffers, excipients), these approaches can significantly enhance product stability. Incorporating deamidation risk assessments early in drug development is increasingly essential. Additionally, advanced delivery systems and formulations designed to resist chemical degradation may further extend the therapeutic potential of protein-based drugs.

## Figures and Tables

**Table 1:** Human neurodegenerative diseases linked to protein deamidation, abnormal misfolding, aggregation, and the deposition of proteins.

Sr No	Neurodegenerative Diseases	Deamidated Protein	Functional Consequences	References
1.	Alzheimer’s disease	Tau	Increased aggregation, reduced microtubule binding.	[72,73,74,75]
		Human serum albumin, neurofilament light protein (NfL), glial fibrillary acidic protein (GFAP)	Deamidation protein biomarkers for detection of neurodegeneration.	[76]
		Amyloid-beta (Aβ), Tau, Protein S100A9, 4E-BP2 Protein, Na^+^/K^+^-ATPase, Ion-Channel Proteins	Structural changes, functional inactivation, and enhanced aggregation. Impacts neurons and axons.	[12,28,29,56,77]
2.	Parkinson’s disease	α-Synuclein, 4E-BP2 Protein	Aggregation, Impact neurons and axons.	[13,56]
		Ceruloplasmin	Ceruloplasmin in the CSF of PD patients undergoes conformational changes and NGR-motif deamidation, which promote the gain of integrin-binding function. Leads to loss of enzymatic activity, also confers gain of function to Cp.	[78,79]
3.	Huntington’s disease	Huntingtin (HTT)	Abnormal conformation.	[80]
4.	Amyotrophic lateral sclerosis	Superoxide dismutase	Structure destabilization, protein aggregation, toxic oligomer formation.	[81]
5.	Prion diseases, Creutzfeldt-Jakob disease	Prion protein (PrP)	Change in conformation, misfolding.	[82]
6.	Frontotemporal dementia	Tau, TDP-43	Neuronal loss.	[74]
7.	Spinocerebellar ataxia	Ataxin (varies by type)	Alter the protein’s stability, folding, and interactions.	
8.	Multiple system atrophy	α-Synuclein	Misfolding and protein aggregation.	[83]
9.	Progressive supranuclear palsy	Tau	Misfolding, aggregation, reduced microtubule binding, acceleration of NFT formation and disease progression.	[75,84]
10.	Corticobasal degeneration	Tau	Misfolding, aggregation, reduced microtubule binding.	[75,84]
11.	Cataract	α-Crystallin, β-crystallin, γ-crystallin	Altered structure, dimer formation, protein aggregation.	[31,62,85]
12.	Lewy body dementia	α-Synuclein	Aggregation.	[13,86]
13.	Vascular dementia	amyloid β peptides, Synapsin1, α-tubulin 1B (TUBA1B) and β-tubulin 2A (TUBB2A) proteins, human serum albumin, Na+/K+-ATPase, ion-channel proteins	Functional impairment and synaptic impairment.	[3,76,77,87,88]
14.	Familial Alzheimer’s disease	Amyloid-beta (Aβ), Tau	Oligomerization/fibrillization, amyloid-related neurodegeneration.	[77,89]
15	Charcot–Marie–Tooth disease	Peripheral myelin proteins (PMP22)	Intracellular aggregation.	[90]
16.	Niemann-Pick disease	Sphingomyelinase	Alterations in the function of the lysosomal system.	[91]
17	Diabetes (type 2 diabetes)	Amylin, islet amyloid polypeptide	Accelerates amyloid formation.	[92,93]
18	Alzheimer’s disease with tauopathy	Tau	Increased rates of β-sheet transition and fibril formation.	[94]
19	Gerstmann–Sträussler–Scheinker syndrome	Prion protein (PrP)	Aggregation and formation of PrP amyloid. Misfolding and pathogenicity of prion proteins.	[82,95]

## Data Availability

Not applicable.
